# Hyaluronic acid and epidermal growth factor improved the bovine embryo quality by regulating the DNA methylation and expression patterns of the focal adhesion pathway

**DOI:** 10.1371/journal.pone.0223753

**Published:** 2019-10-29

**Authors:** Mohammed Saeed-Zidane, Dawit Tesfaye, Yousri Mohammed Shaker, Ernst Tholen, Christiane Neuhoff, Franca Rings, Eva Held, Michael Hoelker, Karl Schellander, Dessie Salilew-Wondim

**Affiliations:** 1 Institute of Animal Science, Department of Animal Breeding and Husbandry, University of Bonn, Bonn, Germany; 2 Animal and Poultry Physiology Department, Animal and Poultry Production Division, Desert Research Center, Mataria, Cairo, Egypt; 3 Teaching and Research Station Frankenforst, Faculty of Agriculture, University of Bonn, Königswinter, Germany; 4 Center of Integrated Dairy Research, University of Bonn, Bonn, Germany; University of Bonn, Institute of Experimental Hematology and Transfusion Medicine, GERMANY

## Abstract

Focal adhesion pathway is one of the key molecular pathways affected by suboptimal culture conditions during embryonic development. The epidermal growth factor (EGF) and hyaluronic acid (HA) are believed to be involved in the focal adhesion pathway function by regulating the adherence of the molecules to the extracellular matrix. However, regulatory and molecular mechanisms through which the EGF and HA could influence the embryo development is not clear. Therefore, this study aimed to investigate the effect of continued or stage specific supplementation of EGF and/or HA on the developmental competence and quality of bovine preimplantation embryos and the subsequent consequences on the expression and DNA methylation patterns of genes involved in the focal adhesion pathway. The results revealed that, the supplementation of EGF or HA from zygote to the blastocysts stage reduced the level of reactive oxygen species and increased hatching rate after thawing. On the other hand, HA decreased the apoptotic nuclei and increased blastocyst compared to EGF supplemented group. Gene expression and DNA methylation analysis in the resulting blastocysts indicated that, combined supplementation of EGF and HA increased the expression of genes involved in focal adhesion pathway while supplementation of EGF, HA or a combination of EGF and HA during the entire preimplantation period changed the DNA methylation patterns of genes involved in focal adhesion pathway. On the other hand, blastocysts developed in culture media supplemented with EGF + HA until the 16-cell stage exhibited higher expression level of genes involved in focal adhesion pathway compared to those supplemented after the 16-cell stage. Conversely, the DNA methylation level of candidate genes was increased in the blastocysts obtained from embryos cultured in media supplemented with EGF + HA after 16-cell stage. In conclusion, supplementation of bovine embryos with EGF and/or HA during the entire preimplantation period or in a stage specific manner altered the DNA methylation and expression patterns of candidate genes involved in the focal adhesion pathway which was in turn associated with the observed embryonic developmental competence and quality.

## Introduction

Suboptimal culture conditions during preimplantation period can result in long-term effects on embryo competence and pregnancy establishment. Despite many attempts to modulate the *in vitro* culture conditions to mimic the *in vivo* environment, the quality of *in vitro* produced embryos remains low [1]. Furthermore, suboptimal embryo culture condition decreases the quality and hinders the developmental competence of the embryo by altering the expression and DNA methylation patterns of developmentally related genes and pathways [2, 3]. Among these, focal adhesion pathway was one of the top dysregulated pathways in bovine embryos due to suboptimal culture conditions [4]. Focal adhesion is one of the cell communication mechanisms, it is vital for cell motility, differentiation, migration, proliferation and survival [5, 6]. Many of focal adhesion proteins, such as beta1 integrin, alpha4 integrin, alpha5 integrin, talin, paxillin, vinculin, focal adhesion kinase and integrin like kinase are essential for embryonic development. Furthermore, the functional loss of these proteins during embryogenesis would affect the cell-extracellular matrix (ECM) adhesion, cytoskeletal organization, polarity, migration and survivability of the embryos [7].

Adhesion to the ECM with the supplementation of growth factors is necessary for normal cell growth [8]. Moreover, supplementation of growth factors in cell-free culture media was found to improve the blastocyst rate [9]. Among these factors, epidermal growth factor (EGF) was found to improve the embryonic development in mouse [10], porcine [11] and bovine [12]. Similarly, hyaluronic acid (HA), one of the main components of ECM, is also believed to improve the blastocyst rate of *in vitro* produced bovine embryos [13]. However, the molecular mechanisms through which supplementation of EGF and HA influenced the embryonic development and quality remain elusive. Here, it was hypothesized that the extracellular growth factors and the extracellular components could improve the quality and development of embryos by regulating the DNA methylation and expression patterns of genes involved in focal adhesion pathway. Indeed, suboptimal *in vitro* culture conditions could cause DNA methylation changes during embryo development [14, 15]. We assumed that, the dynamic changes in the DNA methylation pattern of embryos during *in vitro* development may rely on epigenetic adaptability of embryos resulting from the persistent cellular interactions with extracellular environment via cell adhesion to ECM molecules. Therefore, this study was conducted to investigate the effect of continued or stage specific supplementation of epidermal growth factor and/or hyaluronic acid on the expression and DNA methylation patterns of the genes involved in focal adhesion pathway and its subsequent significance on the developmental competence and quality of bovine preimplantation embryos.

## Materials and methods

### Experimental design

**Fig 1:**
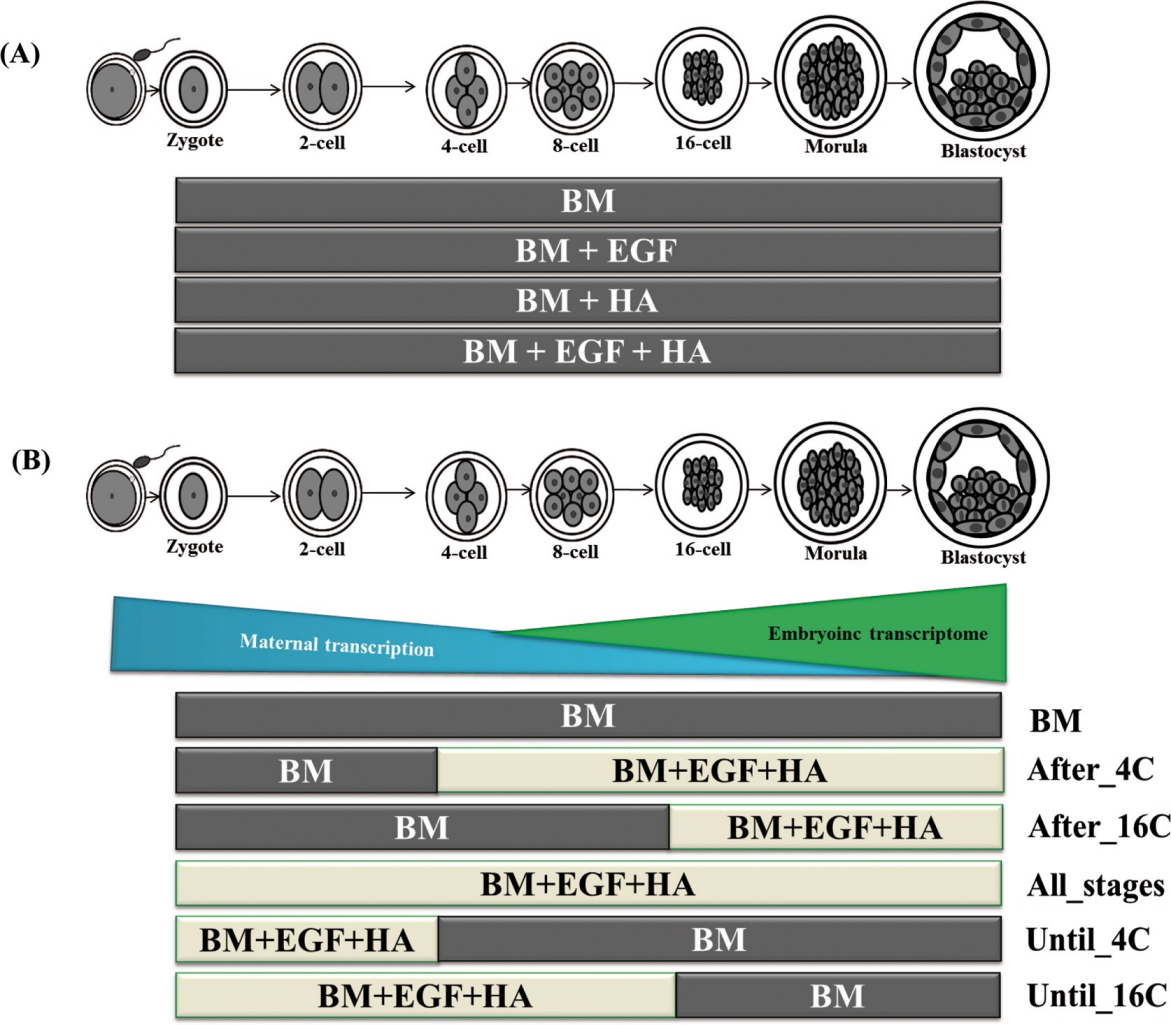
Outline of the experimental design. In first experiment (A), zygotes were cultured in four culture conditions until the blastocysts stage: only in basic media (BM), BM supplemented with EGF, BM supplemented with HA and BM supplemented with EGF + HA. In the second experiment (B), preimplantation embryos were cultured in BM until the blastocyst stage (BM), or supplemented with EGF + HA after 4 cell stage (After_4C), after 16 cell stage (After_16C), until 4-cell (Until_4C), until 16-cell stage (Until_16C) or from zygote to the blastocyst stage (All_stages).

Two consecutive experiments were conducted to achieve the goals of the study. The first experiment was designed to investigate the effect of EGF and/or HA on the expression and DNA methylation patterns of genes involved in focal adhesion pathway and the subsequent effects on the development and quality of bovine embryos. Therefore, supplementation of EGF and/or HA was performed from zygote to the blastocyst stage (the entire preimplantation “period”) For this, synthetic oviduct fluids (SOF) culture media supplemented with fatty acid free bovine serum albumin was considered as the basic media (BM). Accordingly, *in vitro* produced presumptive zygotes were cultured only in BM (control group), or BM supplemented with 10 ng/ml EGF [16–19], BM supplemented with 1mg/ml HA [13, 20–22] or BM supplemented with a combination of 10 ng/ml EGF and 1 mg/ml HA (EGF + HA), (Fig 1A). Afterwards, the effects of EGF and/or HA on the developmental competence of the day 7 preimplantation embryos were investigated. Moreover, blastocysts (four biological replicates) were subjected to total cell count, ICM/TE ratio, ROS accumulation, apoptosis, protein analysis and cryotolerance (three biological replicates) assays. Furthermore, Total RNA and genomic DNA were isolated from each four biological replicates of day 7 blastocysts group. Thereafter, the mRNA expression was quantified using qPCR and the DNA methylation level of candidate genes involved in focal adhesion pathway was analyzed using bisulfite sequencing.

The second experiment, was aimed to elucidate the effect of continued (the entire preimplantation “period”) or stage specific (before or after embryonic genome activation) supplementation of EGF + HA on the expression and DNA methylation patterns of genes involved in focal adhesion pathway (Fig 1B). Accordingly, preimplantation embryos were then cultured in six culture conditions and six blastocyst groups were generated for development assessment and molecular analysis. The first blastocyst group was generated from zygotes cultured in basic media (BM), while the second blastocyst group (After_4C) was generated from zygotes culture in BM until 4-cell stage and then transferred to culture media supplemented with 10 ng/ml EGF and 1 mg/ml HA (EGF + HA) until blastocyst stage. The third blastocyst group (After_16C) was derived from zygotes cultured until 16-cell stage in BM then cultured in media supplemented with EGF + HA until the blastocyst stage. On the other hand, the fourth blastocyst group (All_stages) was obtained from zygotes cultured until blastocyst stage in culture media supplemented with EGF + HA. The fifth group (Until_4C) was obtained from zygotes cultured in media supplemented with EGF + HA until 4-cell stage and further cultured until the blastocyst stage in BM while, the sixth blastocyst group (Until_16C) was generated from zygotes cultured in media supplemented with EGF + HA until the 16-cell stage and then cultured in BM until blastocyst stage. Accordingly, in the second experiment three embryo groups were cultured with media supplemented with EGF + HA during the embryonic genome activation period (All_stages, After_4C and Until_16C) however, three groups were cultured with the absence of EGF + HA during the embryonic genome activation period (BM, Until_4C and After_16C). Accordingly, the development data were recorded and four biological replicates of each blastocysts group (10 blastocysts / replicate) were subjected mRNA expression and DNA methylation patterns of candidate genes involved in focal adhesion were analyzed.

### Oocytes collection and *in vitro* maturation

Bovine ovaries were collected from local slaughterhouse (Duren, Germany) and transported to the laboratory within 1–2 hr in a thermal container containing physiological saline solution (0.9% NaCl) at 37°C. After arrival, the ovaries were washed with saline solution and rinsed in 70% ethanol followed by two washes with saline solution. Cumulus oocyte complexes (COCs) were then aspirated from follicles 2–8 mm in size using a 5-ml syringe loaded with an 18- gauge needle. The COCs with more than three compacted cumulus layers and evenly granulated cytoplasm were cultured in groups of 50 in 400 μl of standard maturation medium [tissue culture media (TCM-199) (M-2154; Sigma, Munich, Germany) with Earle salts buffered with 4.43 mM HEPES (H-9136; Sigma, Munich, Germany) and 33.9 mM sodium bicarbonate (S-5761; Sigma, Munich, Germany) supplemented with 12% estrous cow serum (OCS), 0.5 mM L-glutamine, 0.2 mM pyruvate, 50 mg/ml gentamycin sulphate and 10 μg/ml FSH (Folltropin, Vetrepharm, Canada)) in four well dishes (Nunc, Roskilde, Denmark). The COCs were incubated for 22 hr at 38.7°C and 5% CO_2_ in air with maximum humidity.

### *In vitro* fertilization and embryo production

A group of 50 matured COCs (n = 3550) were *in vitro* fertilized with 2 × 10^6^ spermatozoa/ml using a commercial frozen-thawed semen (Borken, Germany) in Nunclon dishes in 250 μl modified Tyrode medium supplemented with 10 mM sodium lactate, 1 mM sodium pyruvate, 6 mg/ml bovine serum albumin (BSA), 1 μg/ml heparin, 10 μM hypotaurine, 20 μM penicillamine and 2 μM epinephrine at 38.7°C. The COCs and sperms were incubated in 5% CO_2_ in air with maximum humidity. Eighteen hours later, the presumptive zygotes were denuded by repeated pipetting, washed three times in PBS and then cultured (n = 50) in 400 μl of SOF culture medium supplemented with or without EGF and/or HA according to the experimental design (Fig 1) in four well dishes (Nunc, Roskilde, Denmark) covered with mineral oil at 38.7°C and 5% CO_2_ in humidified air. The cleavage and blastocyst rates, blastocyst cell number, apoptotic index, level of reactive oxygen species (ROS) and cryotolerance ability of the embryos were recorded in each treatment group of the first experiment. Moreover, in the second experiment, the cleavage rate and day 7, day 8 and day 9 blastocyst rates were recorded and the day 7 blastocyst stage embryos in both experiments were used for gene expression and DNA methylation analysis.

### ROS accumulation assay

Intracellular ROS levels in four biological replicates of day 7 blastocyst stage embryos (20 blastocysts / replicate) was determined using the cell-permeant 2', 7’-dichlorodihydrofluorescein diacetate (H_2_DCFDA) (Life Technologies, USA) following the manufacturer’s instruction. Briefly, the four independent biological replicates of blastocysts from each group were incubated in 400 μl media containing of 5 μM H2DCFDA for 20 min at 37°C followed by washing twice with PBS-PVA 0.01%. Thereafter, the ROS level was evaluated under inverted fluorescence microscope (Leica DM IRB, Wetzlar, Germany). Afterwards, the images were captured using the green fluorescence filter which emitting 517–527 nm wave length for ROS. The background was adjusted with the negative control then the positive samples were checked using the same adjustments. The image signals were quantified using ImageJ 1.48v software (National Institutes of Health, USA, http://imagej.nih.gov).

### Blastocyst total cell number and the inner cell mass (ICM) and trophoectoderm (TE) differentiation assessment

The day 7 blastocyst total cell number of four independent biological replicates of each group (20 blastocysts / replicate) was determined using Hoechst 33342 stain (Sigma, Munich; Germany). Blastocysts from each group were incubated for 5 min in a solution containing 2% formalin and 0.25% gluteraldehyde. Fixed blastocysts were then mounted and stained with 12.5 μg/ml solution of glycerol-based Hoechst 33342 on clean glass slides for 10 min. Stained nuclei were visualized using the epifluorescent microscope (Olympus, Tokyo, Japan), and then the cells number was recorded. Differential inner cell mass and trophoectoderm cells count was performed according to work described previously Handyside and Hunter [23]. Briefly, the blastocysts (20 blastocysts / group) were subjected to zona pellucida removal by treatment with acid tyrode (pH 2.5). Thereafter, zona-free blastocysts were incubated for 30 min at 39°C in a 1:10 dilution of rabbit antibody (Sigma, D-9656; Germany). Blastocysts were incubated for 30 min at 39°C in a solution of complement (guinea pig complement, sigma, Germany), propidiumiodide (Sigma, Germany) and 8% (v/v) FCS in PBS. Afterwards, the blastocysts were fixed in cold ethanol with bisbenzimide stain (Hoechst 33258, Sigma B2883) and kept for 20 hr at -20°C. Fixed blastocysts were then transferred to a drop of glycerol on a glass slide and covered with a cover slip and the cells were counted under a fluorescent microscope (Leica DM-IRB, Germany).

### Terminal deoxynucleotidyl transferase (TdT)-mediated dUTP-biotin nick end labelling (TUNEL) assay

TUNEL assay kit (Roche^®^, Mannheim, Germany) was used to detect dead cells in day 7 blastocysts of different treatment groups. Four independent biological replicates blastocysts (10 blastocysts / group) from each group were fixed, permeabilized and then incubated with drops of TUNEL solutions for 1 hr in a humid chamber at 37°C in dark. Samples used as positive control were treated with DNase (Promega, WI; USA) while, the negative control ones were incubated with making solution. Thereafter, samples were washed and stained with Hoechst 33342 (Sigma, Munich; Germany), then mounted with glycerol on clean slides. The samples were observed under the fluorescence microscope (Leica, Germany) and TUNEL positive (fragmented DNA) cells were recorded.

### Cryotolerance test

To investigate the effect of EGF and/or HA on embryo freezability, three biological replicates of the blastocyst stage embryos cultured in BM (n = 67), BM + EGF (n = 59), BM + HA (n = 120) and BM + EGF + HA (n = 63) were subjected to the cryotolerance test. Briefly, the day 7 blastocysts were washed with D-PBS containing 5% PVA and then transferred to a solution consisting of 1.5 M ethylene glycol and 0.1 M sucrose (BoviFreeze, Minitube GmbH, Tiefenbach, Germany). Groups of 10–15 blastocysts were loaded into an open-pulled straw (Minitube GmbH, Tiefenbach, Germany) and then immediately plunged into the programmable freezer “Freeze Control” (Consarctic, Germany). Seeding was accomplished manually with forceps cooled in liquid nitrogen at a temperature of -6°C, followed by a cooling to -30°C at a rate -0.3°C /min. Thereafter, the straws were kept in liquid nitrogen. One week later, the embryos were thawed and washed with SOF media supplemented with fatty acid free BSA. Finally, the embryos were cultured in 400 μl culture media in four well dishes (Nunc, Roskilde, Denmark) covered with mineral oil at 38.7°C in 5% CO_2_ and humidified air. The expansion and hatching rates were determined at 24, 32, 48 and 56 hr post thawing.

### DNA and total RNA isolation and first strand cDNA synthesis

The genomic DNA (gDNA) and total RNA of each experimental group were isolated from four biological replicates of day 7 blastocysts (10 blastocysts / replicate) using RNA/DNA/protein purification plus micro kit (Norgen biotek, Cat# 51600, Canada) according to manufacturer’s instructions. The gDNA was used for DNA methylation analysis whereas the total RNA was used for the gene expression studies. Total RNA concentration was measured using NanoDrop 8000 spectrophotometer (NanoDrop technologies, USA) and the adjusted RNA (100 ng) was reverse transcribed into cDNA using thermo scientific first strand cDNA synthesis kit (Life technologies, GmbH, Germany). Briefly, total RNA of each sample was incubated with 0.5 μl oligo-dT and 0.5 μl random primers at 65°C for 5 min followed by chilling on ice. Afterwards, 1 μl ribolock RNase inhibitor, 4 μl 5x reaction buffer, 2 μl of 10 mM dNTPs mix and 2 μl reverse transcriptase were added and incubated at 25°C for 5 min, 37°C for 60 min followed by 70°C for 5 min. Samples were then stored at -20°C until use.

### Gene expression analysis

To determine the effect of EGF and/or HA supplementation during *in vitro* embryo culture on the expression of genes involved in focal adhesion pathway, the mRNA level of candidate genes was determined using quantitative real time PCR (qPCR). For that, the mRNA expression level of 11 genes namely, actin gamma1 (ACTG1), focal adhesion kinase (FAK), phosphatase and tensin homolog (PTEN), vinculin (VCL), p21 protein (Cdc42/Rac)-activated kinase 4 (PAK4), ras-related C3 botulinum toxin substrate 1 (RAC1), collagen, type IV, alpha 1 (COL4A1), collagen, type I, alpha 2 (COL1A2), epidermal growth factor receptor (EGFR), CD44 molecule (CD44) and hyaluronan-mediated motility receptor (HMMR) were selected for analysis. In addition, the expression level of DNA (cytosine-5-)-methyltransferase 1 (DNMT1), DNA (cytosine-5-)- methyltransferase 3 alpha (DNMT3A) and DNA (cytosine-5-)-methyltransferase 3 beta (DNMT3B) was also investigated. For all samples 2 μl cDNA of four biological replicates were used and the qPCR was performed using Applied Biosystem® StepOnePlus™ System (Thermo Fisher Scientific Inc, USA) using iTaq™ Universal SYBR® Green Supermix (Bio-Rad Laboratories GmbH, Germany) with the following program; 95°C for 3 min, 40 cycles at 95°C for 15 sec, 60°C for 45 min. The data from four independent biological replicates were analyzed using a comparative threshold cycle method (2^-ΔΔCT^) using beta actin (β-actin) and glyceraldehyde phosphate dehydrogenase (GAPDH) expression as the normalizers. All primers (Table 1) used for qPCR were designed using the online software (http://www.ncbi.nlm.nih.gov/tools/primer-blast/).

**Table 1:** List of primers used for quantitative real time PCR analysis.

Accession number	Genes	Primer sequence	Size
NM_173979	ACTB	F: 5´-GGCATTCACGAAACTACCTT-3´ R: 5´-CAATCCACACGGAGTACTTG-3´	208
NM_001034034	GAPDH	F: 5´-ACCCAGAAGACTGTGGATGG-3´ R: 5´-ACGCCTGCTTCACCACCTT-3´	247
NM_001033618	ACTG1	F: 5´-ATCCGAAAGGACCTGTATGC-3´ R: 5´-CTTGCTGATCCACATCTGCT-3´	213
NM_001075250	FAK	F 5´-ACTGGATTCAGTCAAGGCCA-3´ R 5´-CAGCCCTTGTCTGTGAGGTA-3´	231
XM_613125	PTEN	F:5´-TGGGGAAGTAAGGACCAGAG-3´ R: 5´-ATTGCAAGTTCCACCACTGA-3´	172
NM_001191370	VCL	F:5´-TTGCAAAGTGTGACCGAGTG-3´ R: 5´-CAACAGCTTGATGGGAGTCG-3´	203
NM_001076184	PAK4	F: 5´-TACCAGCATGAGAACGTGGT-3´ R: 5´-CTCTTGATGTCCCGGTGGAT-3´	212
NM_174163	RAC1	F: 5´-ACGGAGCTGTGGGTAAGACT-3´ R: 5´-TACATCTGTCTGCGGGTAGG-3´	200
NM_001166511	COL4A1	F: 5´-TCTGGATCGGCTACTCCTTT-3´ R: 5´-AACATCTCGCTCCGCTCTAT-3´	196
NM_174520	COL1A2	F: 5´-TGAAAAAGGTCATGCTGGTC-3´ R: 5´-TACCCCTTTCTCCTGGTTTG-3´	200
HM749883	EGFR	F: 5´-GACCCGAAAGAACTGGACAT-3´ R: 5´-TGTTATATCCAGGCCGACAA-3´	177
NM_174013	CD44	F: 5´-CTGAAATGAGGGCCCAGTTA-3´ R: 5´- CCAACCCCACTTGAAAGAAA-3´	285
NM_001206621	HMMR	F: 5´-TGCTTATACTCAGGCCACCC-3´ R: 5´-CGGACATCCTCTGCACTTTG-3´	189
NM_182651	DNMT1	F: 5´-TGACTACATCAAGGGCAGCA-3´ R: 5´-AGGTTGATGTCTGCGTGGTA-3´	192
NM_001206502	DNMT3B	F: 5´-CTGCTGAATTACACTCGCCC-3´ R: 5´-CCAGAAGTATCGGGCTCTGT-3´	177
NM_181813	DNMT3A	F: 5´-AGCACAACGGAGAAGCCTAA-3´ R: 5´-CAGCAGATGGTGCAGTAGGA-3´	245

### Immunoblotting

Western blot was performed to detect the expression levels of focal adhesion pathway marker proteins (FAK and VCL) in embryos derived from zygotes cultured in media supplemented with or without EGF and/or HA. For this, (30 embryos / replicate) of day 7 blastocysts from each treatment group were boiled with 4 μl 2x SDS loading buffer at 95°C for 5 min and loaded on 4–18% gradient SDS-PAGE gel. After electrophoresis, proteins were transferred to nitrocellulose membrane (Protran®, Schleicher & Schuell Bioscience) and the later was then blocked with Roti-block solution (Carl Roth GmbH) for 1 hr at room temperature. The membrane was overnight incubated at 4°C with anti FAK goat polyclonal antibody diluted at 1:250 (Santa Cruz Biotechnology Inc, Germany). On the second day, the membrane was washed with Tween-Tris-buffer saline (TTBS) and incubated with donkey anti goat secondary antibody diluted at 1:5000 (Santa Cruz Biotechnology Inc, Germany) for 1 hr at room temperature. Thereafter, the membrane was washed with TTBS and incubated in dark with an equal amount of peroxide solution and luminal enhancer for 5 min at room temperature. Images were developed on the ChemiDoc™ XRS+ system (Bio-Rad Laboratories GmbH, Germany). Afterwards, the membrane was subjected to stripping protocol (Bio-Rad, Germany), followed by blocking with the Roti-block solution (Carl Roth GmbH). The membrane was then incubated overnight at 4°C with anti VCL rabbit polyclonal antibody (1:250) and after washing steps it incubated for 1 hr at room temperature with goat anti rabbit secondary antibody diluted at 1:5000 (Santa Cruz Biotechnology Inc, Germany). Afterwards the protein image was detected and the membrane was subjected to stripping and blocking procedure and then incubated overnight at 4°C with anti ACTB mouse monoclonal antibody (1:500) (Santa Cruz Biotechnology Inc, Germany) followed by washing steps and incubation for 1 hr at room temperature with goat anti mouse secondary antibody diluted at 1:5000 (Santa Cruz Biotechnology Inc, Germany) then subjected to image detection process.

### Immunohistochemistry

The localization of VCL protein was performed using an immunohistochemistry assay. Briefly, four biological replicates (15 blastocysts / replicate) from each group were washed three times in phosphate- buffer saline (PBS), and then fixed overnight at 4°C in 4% paraformaldehyde in PBS. Fixed samples were washed twice with glycine in PBS and then permeabilized with 0.5% (v/v) Triton-X100 (Sigma, Munich, Germany) in PBS for 4 hr at room temperature. The permeabilized blastocysts were incubated in 3% normal donkey serum (Sigma, Munich, Germany) in PBS for 1 hr at room temperature. Samples were incubated overnight at 4°C with anti VCL rabbit polyclonal antibody diluted at 1:250 (Santa Cruz Biotechnology Inc, Germany). Afterwards, blastocysts were incubated at 37°C for 2 hr in the dark with FITC-conjugated goat anti-rabbit secondary antibody diluted at 1:200 (Lifespan Biosciences, Seattle, WA). Blastocysts were mounted in mounting medium containing DAPI on a clean slid glass and were then visualized under the CLSM LSM-780 confocal laser scanning microscope (Zeiss, Germany). For that, the confocal microscope was adjusted using the suitable wavelength for FITC (excitation/emission, 495/517) and DAPI dyes at 40x magnification then the background was adjusted using the negative control samples (blastocysts incubated with secondary antibody only) thereafter, images were acquired for all samples. The images were analyzed using ImageJ 1.48v software (National Institutes of Health, USA, http://imagej.nih.gov). The images were analyzed separately and the intensity of the signal from each embryo was recorded and the data were subjected to statistical analysis.

### Bisulfite sequencing

The genomic DNA (gDNA) isolated from four biological replicates of day 7 blastocysts was subjected to bisulfite treatment using EZ DNA methylation direct kit (Zymo Research, USA) and amplified using gene specific primers (Table 2) according to the manufacturer’s recommendation. The primers for bisulfite sequencing were designed using the online software (http://www.urogene.org/cgi-bin/methprimer/methprimer.cgi). The PCR amplification was performed using 2 μl of bisulfite treated gDNA, 1 μl of forward primer, 1 μl of reverse primer, 12.5 μl of 2x reaction buffer, 0.3 μl of 10 mM dNTP mix, 0.2 μl of Taq polymerase, Zymo Taq^™^ DNA polymerase (Zymo Research, USA) and nuclease free water was added up to 25 μl and the mix was then incubated in thermocycler using PCR touchdown protocol. The PCR thermocycler was adjusted using touch down program as following 95°C 5 min 1 cycle, 95°C 30 sec and touch down 0.5°C each cycle till reach the annealing TM followed by 72°C 1 min for 10 cycles, 95°C 30 sec, 55°C 30 sec followed by 72°C 1 min for 35 cycles and 72°C 10 min 1 cycle. The presence of the PCR product was confirmed after loading 5 μl of the PCR product on 2% agarose gel electrophoreses run on 1% TE buffer. The remaining PCR product was purified using the QIAquick PCR purification (Qiagen, Germany), cloned to pGEM®-T Easy Vector Systems (Promega, WI, USA) and transformed to *E*. *coli* competent cells. The bacterial culture was then plated onto the LB agar/ampicillin/IPTG/X-gal plate and incubated overnight at 37°C. Independent clear white colonies were selected for sequencing. For that, the colonies were lysed at 95°C for 15 min and 10 μl of the lysate was used for PCR amplification using M13 primer. The samples (10 μl) in addition to 0.5 μl of M13 forward primer, 0.5 μl of M13 reverse primer, 2 μl of 10x reaction buffer, 0.5 μl of 10 mM dNTP mix, 0.2 μl of Taq polymerase and nuclease free water was added up to 20 μl were incubated in thermocycler using the following PCR protocol, 95°C 30 sec, 60°C 30 sec followed by 72°C 1 min for 40 cycles and 72°C 10 min 1 cycle The PCR product was then subjected to sequencing protocol and sequenced using the GenomeLab™ GeXP Genetic Analysis System (Beckman Coulter). The bisulfite sequencing DNA methylation analysis software (BISMA) (http://services.ibc.uni-stuttgart.de/BDPC/BISMA/) was used to analyze the sequencing data.

**Table 2:** List of primers used for DNA methylation analysis.

Accession number	Genes	Primer sequence	Number of CpGs in the product
ENSBTAG00000013472	COL1A2	F: 5´-GGGATTTTAAGTTTATTTTTTAATAAA-3´ R: 5´-AACCCTACCTACCTTATACCCTAC-3´	16
		F: 5´- TGGTTTGTTGGTAAAGTTTATTTTTTT -3´ R: 5´-CCAACCCTACCTACCTTATACCCTAC-3´	22
ENSBTAG00000012849	COL4A1	F 5´-GGGTAGGATTTTATATTAGTTTTTGATGTT-3´ R 5´-ACCTTCTCTTAAATTCCCCTTCAAT-3´	7
		F: 5´-GATAGAGGAGAAGGGGAATTTAGGAT-3´ R: 5´-AACTCCCACCAAAAACCCTATTT-3´	9
ENSBTAG00000009233	RAC1	F 5´-AAGTGAATGGTAGTTTTTAGGAATTT-3´ R 5´-AAAAAAATTATTTACCTCCCATTAATA-3´	8
		F 5´-TTTTTGGGGTTTGGATATTTGTAT-3´ R 5´-AAACCAAACACCCCTAAAACTAAAC-3´	14

### Statistical analysis

The data of this study were analyzed using Statistical Analysis System (SAS) version 9.1 software (SAS Institute Inc., Cary, NC, USA). One-way analysis of variance (ANOVA) followed by Tukey multiple pairwise comparison was performed between treatment groups. Number of blastocysts survived after cryopreservation was analyzed using the chi square test. Differences were considered significant when P < 0.05.

### Ethics approval and consent to participate

The study was conducted on bovine *in vitro* produced blastocysts using oocytes collected from slaughterhouse ovaries and thus special approval of this experiment was not required.

## Results

### Hyaluronic acid supplementation influenced embryo development and apoptotic index

**Fig 2:**
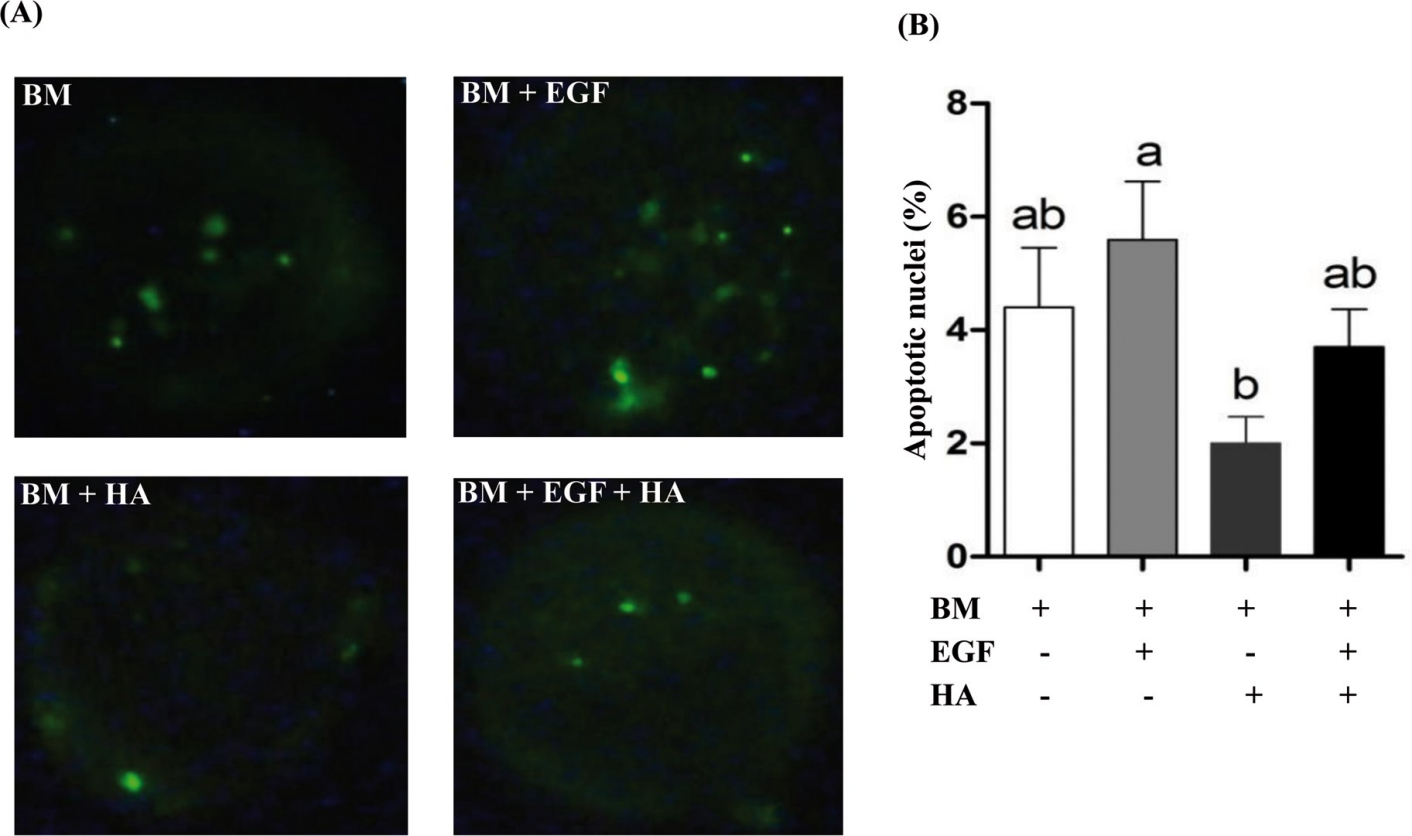
TUNEL assay in blastocysts of different blastocysts group. (A) Representative blastocysts showing TUNEL positive cells. (B) The percentage of apoptotic nuclei is presented as mean ± SEM. Bars with different letters are statistically significant different at *p* < 0.05.

Supplementation of culture media with EGF, HA or EGF + HA did not affect the cleavage rate and blastocyst formation compared to the control group. Nevertheless, the day 7 blastocyst rate was significantly higher in the HA supplemented group compared to the EGF group (Table 3). Moreover, supplementation of HA resulted in insignificant increases in blastocyst total cell number and the ICM: TE ratio (Table 3). On the other hand, supplementation of HA reduced the apoptotic index in the resulting blastocysts compared to EGF counterparts (Fig 2).

**Table 3:** The development rate and blastocysts cell number of embryos cultured in presence of EGF, HA or EGF + HA throughout preimplantation period.

	BM	BM + EGF	BM + HA	BM + EGF + HA
No. zygotes	425	416	466	446
Cleavage rate	81.3 ± 4.9	82.2 ± 5.9	84.4 ± 6.2	82.1 ± 3.8
Day 7 blastocyst rate	28.9 ± 7.1^ab^	23.8 ± 7.08^a^	32.1 ± 6.1^b^	28.7 ± 5.3^ab^
Day 8 blastocyst rate	42.7 ± 8.1	37 ± 8.2	42.1 ± 5.6	38.6 ± 5.6
Day 9 blastocyst rate	46.1 ± 6.4	41.5 ± 7.4	44.9 ± 5.9	39.7 ± 7.4
Blastocyst total cell number	127±7	123±13	131±4	118±6
ICM:TE ratio	1.73	1.74	1.87	1.81

Data represented as mean ± SEM and different letters in the same raw indicate significantly different (p < 0.05.).

### Epidermal growth factor and/or hyaluronic acid supplementation reduced the oxidative stress and increased the cryotolerance ability in the resulting blastocysts

**Fig 3:**
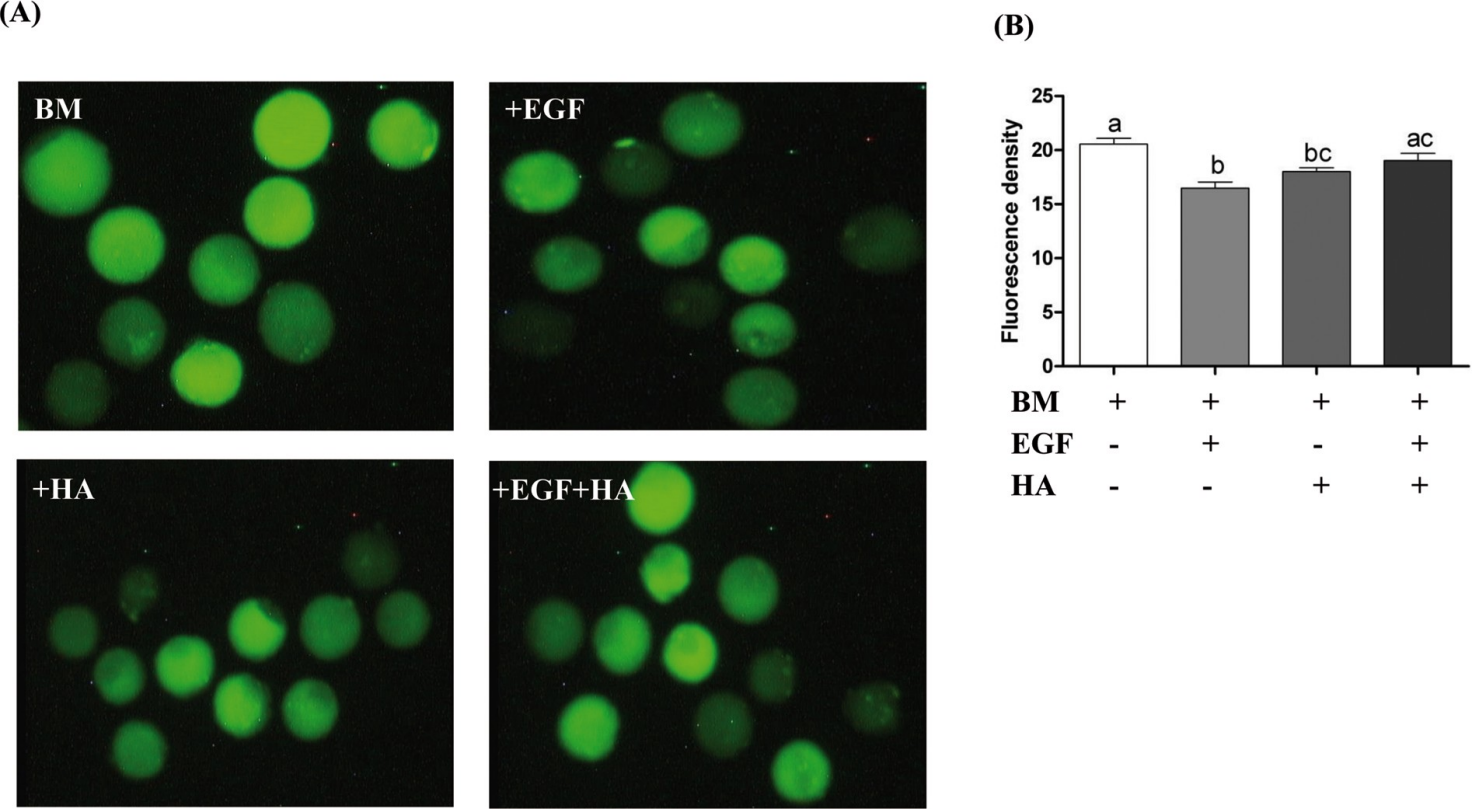
Intracellular ROS accumulation in blastocysts derived from zygotes cultured in media supplemented with EGF and/or HA. (A) Representative figures displaying the ROS accumulation levels in the blastocysts. (B) The fluorescent density (mean ± SEM) showing the level of ROS in each blastocyst group. Bars with different letters are statistically significant (*p* < 0.05). Scale bars represent 100 μm.

**Fig 4:**
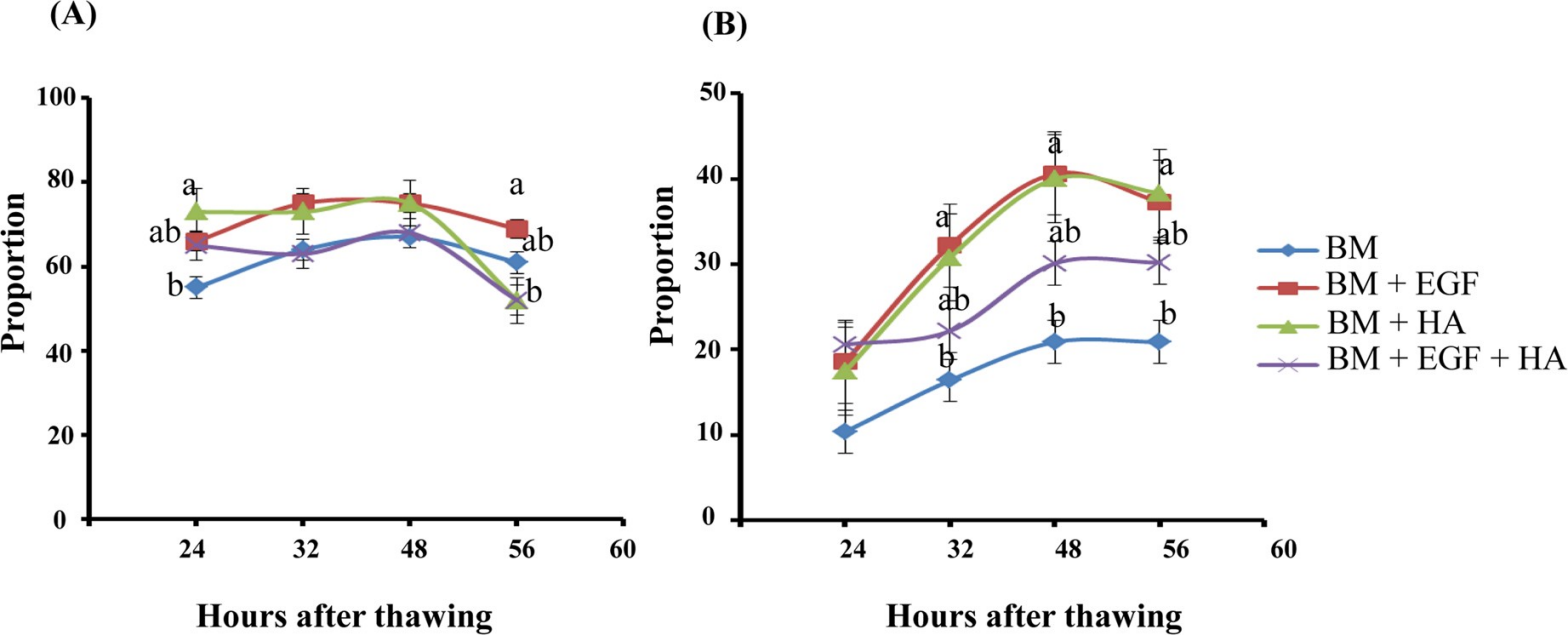
Effects of EGF and/or HA supplementation on the cryotolerance ability of the embryos. Expansion (A) and hatching rates (B) of blastocysts at 24, 32, 48 and 56 hr after thawing of embryos supplemented with EGF and/or HA. Data are presented as mean ± SEM and different letters on the bars indicate statistical significant differences (*p* < 0.05).

In this study, the reactive oxygen species (ROS) levels were significantly reduced in the blastocysts derived from zygotes cultured in culture media supplemented with EGF or HA compared to control ones. However, blastocysts derived from zygotes supplemented with EGF + HA did not show a significant difference in ROS levels compared to control and HA groups (Fig 3). In addition, the blastocysts derived from zygotes cultured in media supplemented with HA exhibited higher re-expansion rates at 24 hr after thawing compared to the control group. However, at 56 hr post thawing, the re-expansion rate was significantly reduced in the HA group compared to the EGF group (Fig 4A). Moreover, the hatching rate was significantly higher in EGF and HA groups at 32, 48 and 56 hr after thawing compared to the control counterparts (Fig 4B).

### Epidermal growth factor and/or hyaluronic acid supplementation altered the expression level of epidermal growth factor and hyaluronic acid receptor genes

**Fig 5:**
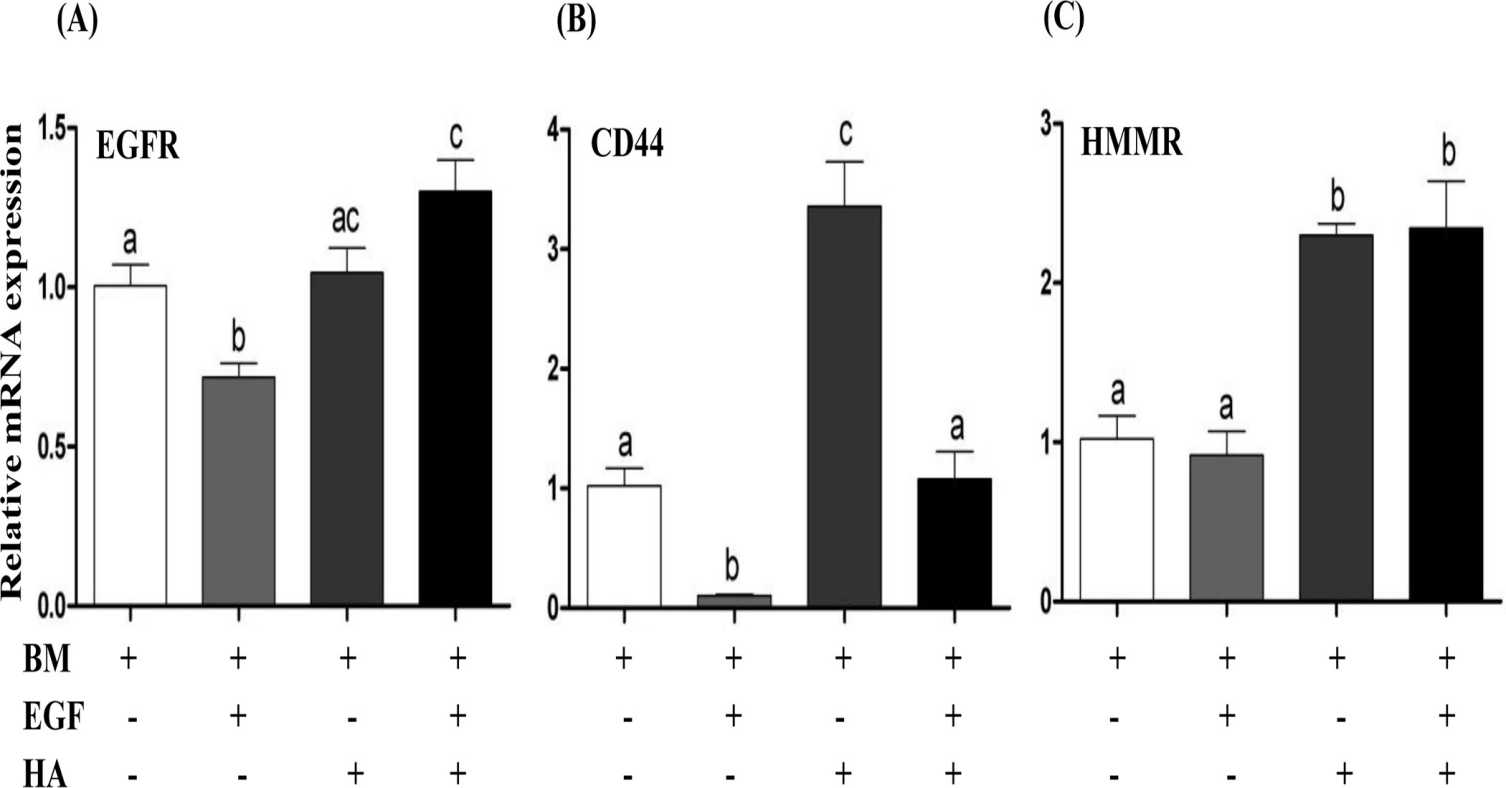
**Supplementation of EGF and/or HA on the expression level of EGF (A) and HA receptors (B and C)**. Data are presented as mean ± SEM. Bars with different letters are statistically significant (*p* < 0.05).

In order to identify the association of EGF and/or HA supplementation and the activity of their receptors, the mRNA abundance level of EGF and/or HA receptors were quantified in blastocysts derived from embryos developed in the culture media supplemented with EGF and/or HA during the entire preimplantation period. While supplementation of HA increased the expression level of its receptor (CD44 and HMMR) genes, supplementation of EGF significantly reduced the mRNA expression level of its receptor (EGFR). However, when EGF was combined with HA, the expression level of EGFR was significantly increased compared to control and EGF groups (Fig 5).

### Epidermal growth factor and hyaluronic acid supplementation induced the expression of genes involved in focal adhesion pathway

**Fig 6:**
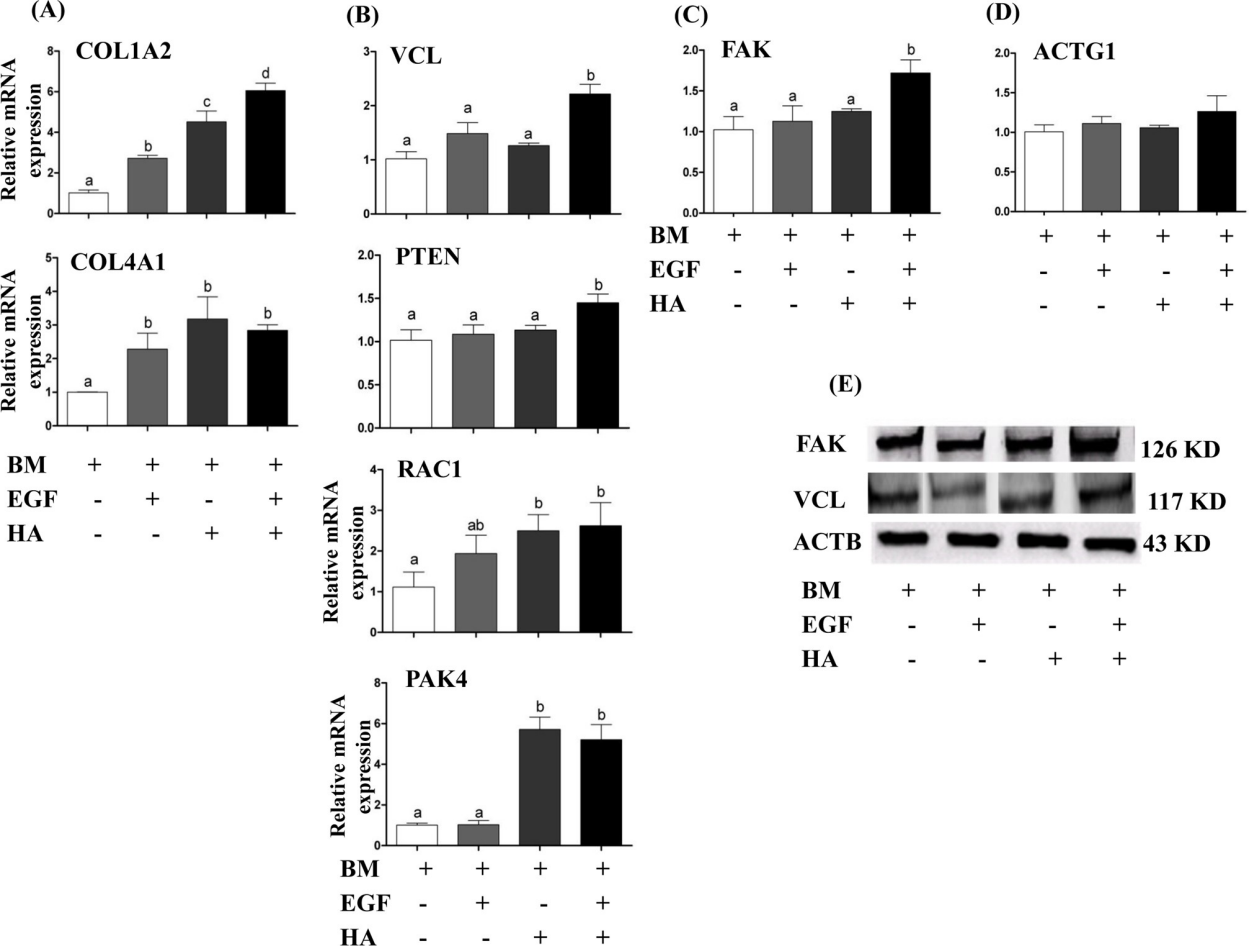
**The mRNA expressions level of ligands** (A), adaptor (B), regulator (C) and structure linker (D) of focal adhesion pathway genes in blastocysts of different treatment groups. (E) Western blot analysis of FAK and VCL proteins in blastocysts derived from zygotes supplemented with or without EGF and/or HA (E). Bars represent mean ± SEM. Different letters on bars are statistically significant (*p* < 0.05).

**Fig 7:**
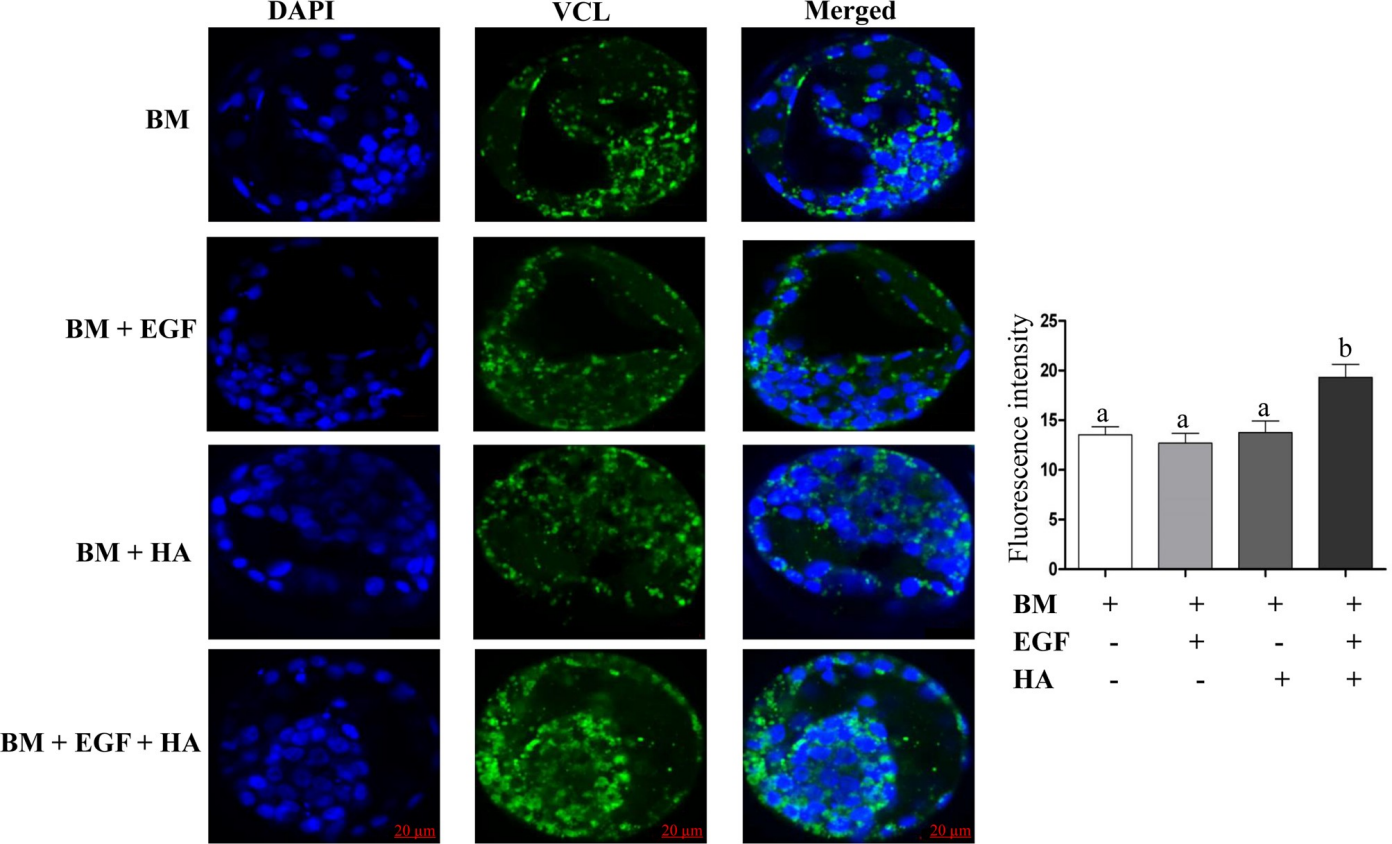
Immunohistochemical analysis of VCL, focal adhesion protein marker gene, in blastocysts derived from zygotes cultured in different treatment groups. The green colour indicates the expression of protein, while the blue colour indicates the nuclear staining using 4′,6-diamidino-2-phenylindole (DAPI).

The expression level of candidate genes involved in focal adhesion pathway was analyzed in blastocysts derived from treatment of different groups. For this, the mRNA level of the genes representing the ligands (COL1A2 and COL4A1), cytoplasmic structures (ACTG1), adaptors and focal adhesion marker (VCL), enzymatic regulation (FAK), inhibitor of focal adhesion kinase (PTEN) and actin regulators (RAC1 and PAK4) was quantified using qPCR. The results revealed that except ACTG1, the expression level of the candidate genes was increased in the blastocysts derived from embryos cultured in the presence of EGF + HA during the entire preimplantation period (Fig 6). Furthermore, immunohistochemical analysis indicated higher VCL protein in the cell membrane of the blastocysts derived from embryos cultured in media supplemented with EGF + HA (Fig 7).

### Combined supplementation of epidermal growth factor and hyaluronic acid altered expression of genes involved in focal adhesion pathway in a stage specific manner

**Fig 8:**
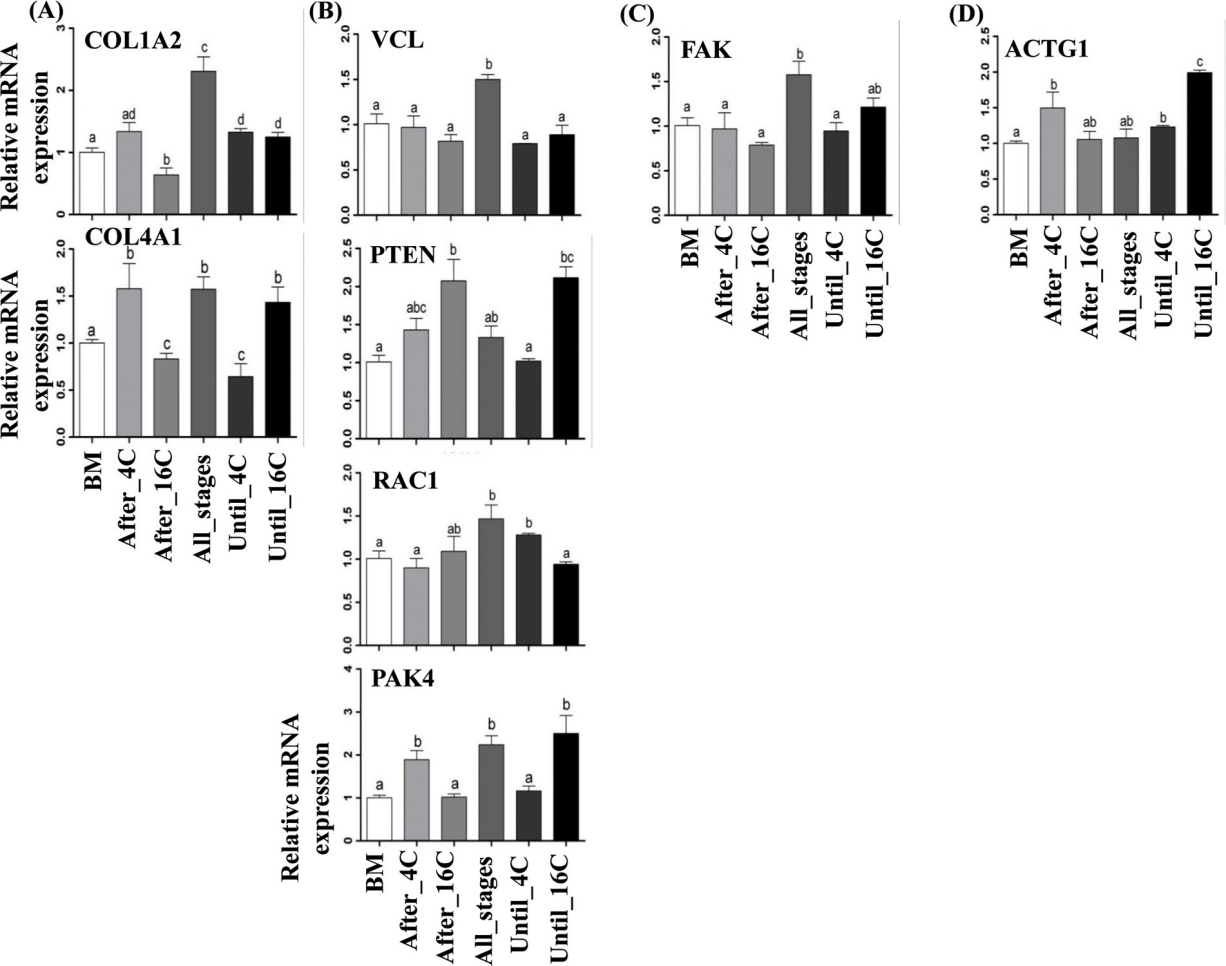
**Supplementation of EGF + HA before or after embryonic genome activation on the expression pattern of ligands (A), adaptor (B), regulator (C) and structure linker (D) of focal adhesion pathway genes**. Data presented as mean ± SEM. Different letters are statistically significant (*p* < 0.05).

The results from the first experiment indicated that the preimplantation embryos cultured from zygote to blastocyst stage in the presence of EGF + HA showed a significant increase in the mRNA and protein expression level of genes involved in focal adhesion pathway. However, in the first experiment, supplementation of EGF and/or HA was performed through the preimplantation period and it was not clear whether the effect of EGF + HA was prominent before, during or after the embryonic genome activation. Therefore, to address this, embryos were supplemented with EGF + HA after 4-cell (After_4C) or after 16- cell stage (After_16C). Other embryo groups were cultured in BM supplemented with EGF + HA until 4-cell (Until_4C) or until 16-cell stage (Until_16C) and then cultured only in BM until the blastocysts stage. Although the number of embryos reached day7 blastocyst stage was higher in Until_16C compared to Until_4C or After_16C while was higher at day8 and day9 blastocyst stage of After_4C group, however, there was absence of significant differences between all experimental groups (Table 4). On the other hand, the gene expression analysis indicated that the expression level of the candidate genes (COL4A1, PAK4and ACTG1) was higher in the blastocysts of After_4C and Until_16C supplemented groups compared to After_16C supplemented group (Fig 8). However, unlike other genes, the expression level of RAC1 was increased in the blastocysts of Until_4C group.

**Table 4:** The correlation between the DNA methylation and expression patterns of the candidate genes in blastocysts of different groups.

Expression = E		After_4C		After_16C		All_stages		Until_4C		Until_16C		BM + EGF		BM + HA	
Methylation = M		E	M	E	M	E	M	E	M	E	M	E	M	E	M
COL1A2	Promoter	↑	*↑*	↓	*↑*	↑	↓	↑	↓	↑	*↑*	↑	*↑*	↑	↓
	Distal Promoter	↑	*↑*	↓	*↑*	↑	*↑*	↑	*↑*	↑	*↑*	↑	*↑*	↑	*↑*
COL4A1	Promoter	↑	↑	↓	↑	↑	↑	↓	↑	↑	↑	↑	↑	↑	─
	Distal Promoter	↑	↓	↓	↓	↑	↑	↓	↑	↑	↓	↑	↓	↑	↓
RAC1	Promoter	─	─	─	↑	↑	↑	↑	↑	─	─	─	↑	↑	↑
	Intron	─	─	─	─	↑	↑	↑	↑	─	↑	─	↓	↑	─

Symbols ↑ and↓ indicate up and downregulation of gene expression, respectively while ↑ and ↓ indicate the hypermethylation and hypomethylation of the gene, respectively.

### Supplementation of epidermal growth factor and/or hyaluronic acid altered the DNA methylation patterns of genes involved in focal adhesion pathway

**Fig 9:**
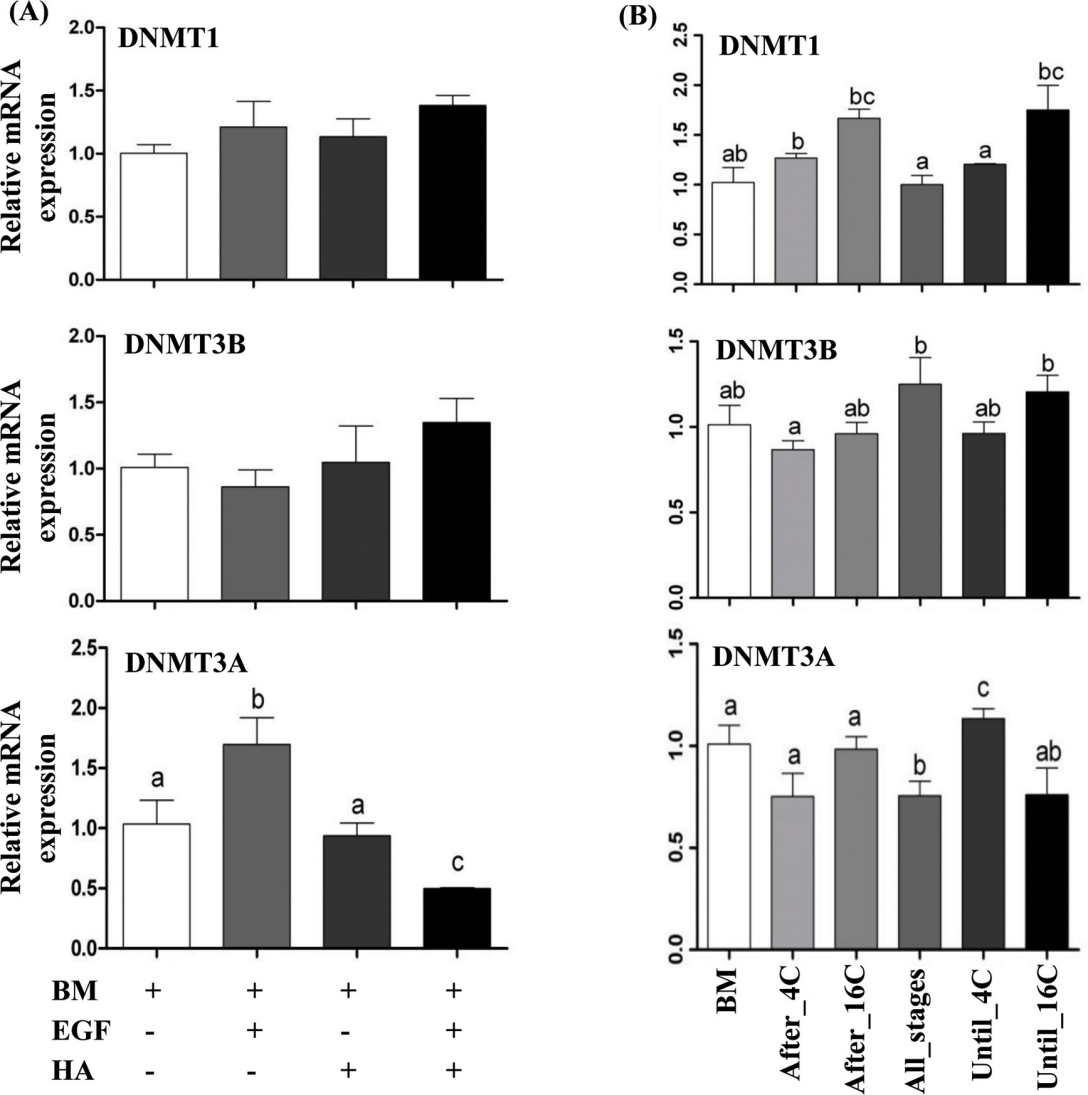
**Relative mRNA expression level of DNMT1, DNMT3B and DNMT3A in blastocysts derived from zygotes cultured in media supplemented with or without EGF and/or HA until blastocyst stage (A) or blastocysts derived from embryo supplemented with or without EGF + HA before or after embryo genome activation (B)**. Data are presented as mean ± SEM. Bars with different letters are statistical significant (*p* < 0.05).

Since supplementation of EGF and/or HA affected both the blastocyst quality and the expression of genes involved in focal adhesion pathway, we speculated that EGF and/or HA may affect the embryo quality by epigenetically regulating the genes involved in focal adhesion pathway. For this, first the expression levels of genes associated with DNA methylation namely, the DNA methyltransferase related genes (DNMT1, DNMT3A and DNMT3B) were investigated in the blastocysts obtained from embryos cultured in media supplemented with EGF, HA and EGF + HA from zygote to blastocyst stage or with EGF + HA before or after embryonic genome activation. Results showed that the expression level of DNMT3A was significantly increased in the blastocysts derived from culture media supplemented with EGF and decreased in the EGF + HA group. However, HA alone or in a combination with EGF did not show any significant effects on the expression levels of DNMT1 and DNMT3B (Fig 9A). Nevertheless, the expression level of DNMT1 was significantly higher in After_4C supplemented group compared to Until_4C supplemented ones. In addition, the expression level of DNMT3B was significantly higher in blastocysts of Until_16C compared to After_4C group, whereas the mRNA level of DNMT3A was significantly increased in the Until_4C supplemented group (Fig 9B).

Following the expression analysis, we have performed DNA methylation analysis in three candidate genes, as their expression was affected by continued or stage specific supplementation of EGF + HA and their critical role in focal adhesion pathway. The three selected genes are namely COL4A1 (Chr. 12, 11623958–11624257 with 7 CpG sites at the promoter region and 9 CpG sites at distal promoter region 11623579–116239544), COL1A2 (Chr. 12, 89010212–89010447 with 16 CpG sites at the promoter region and 22 CpG sites at the distal promoter region 890106686–890106949) and RAC1 (Chr. 25, 38828881–38829079 with 8 CpG sites at the prompter region and 14 CpG sites at the intronic region 38834424–38834685). DNA methylation analysis was performed using bisulfate sequencing from four biological pools of embryos in each experimental group. About 16 clones from each experimental group were subjected for sequencing and 8 clones with accurate sequencing results were subjected for final analysis.

**Fig 10:**
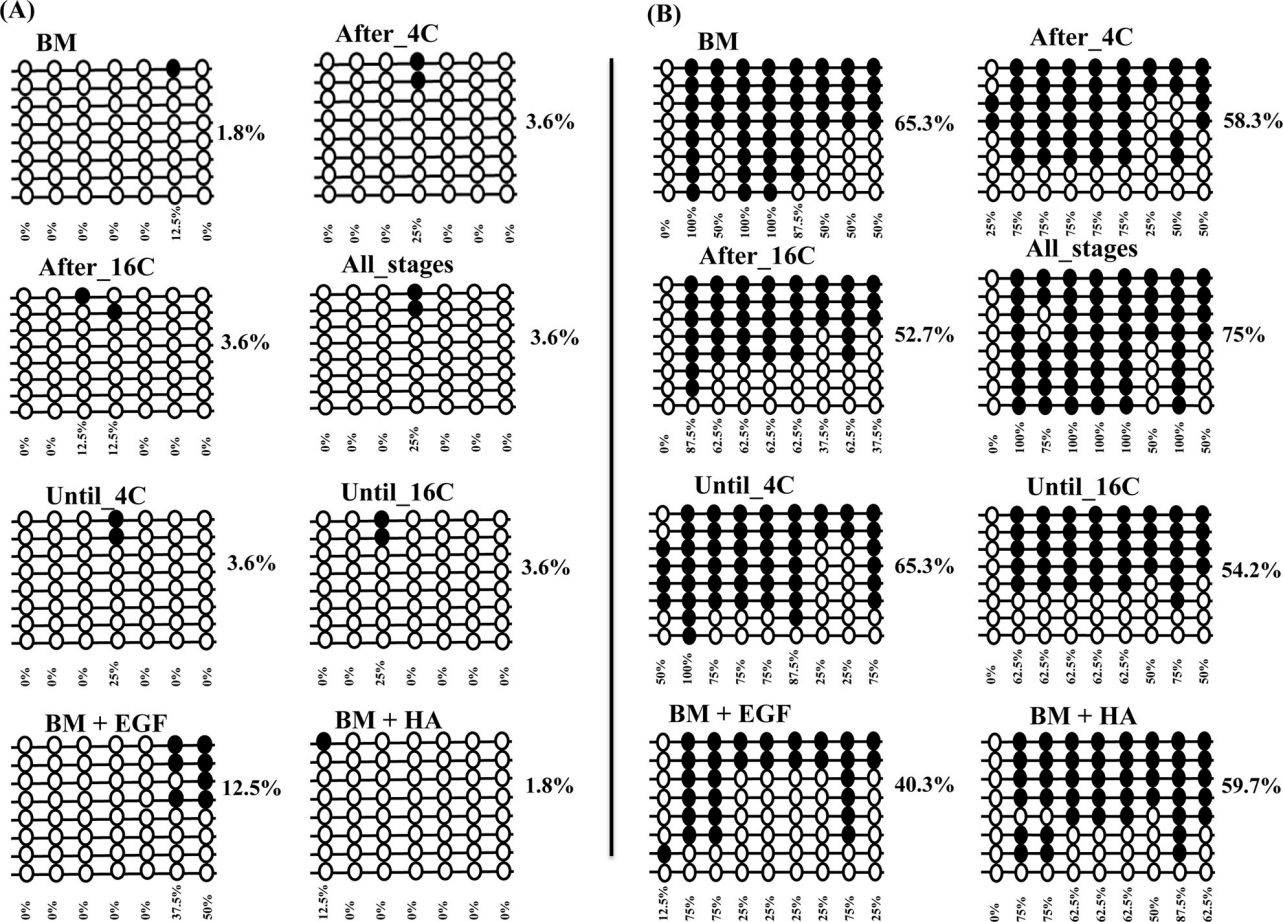
**The DNA methylation level of COL4A1 at the promoter (A) or distal promoter (B) region of blastocysts of different treatment groups**. The rows indicate the sequence of different colonies while, the circles indicate the CpG sites within the sequence. The black and white circles indicate the methylated and unmethylated CpG sites, respectively.

Results revealed that, unlike HA supplemented group, *in vitro* culture of embryos in the presence of EGF alone or in a combination with HA increased and decreased the DNA methylation level of the COL4A1 gene at promoter and distal promoter regions, respectively compared to the control group. On the other hand, except Until_4C group, the DNA methylation level of COL4A1 gene was reduced in the stage specific supplemented groups (EGF + HA before, during or after embryo genome activation) compared to control one (Fig 10).

**Fig 11:**
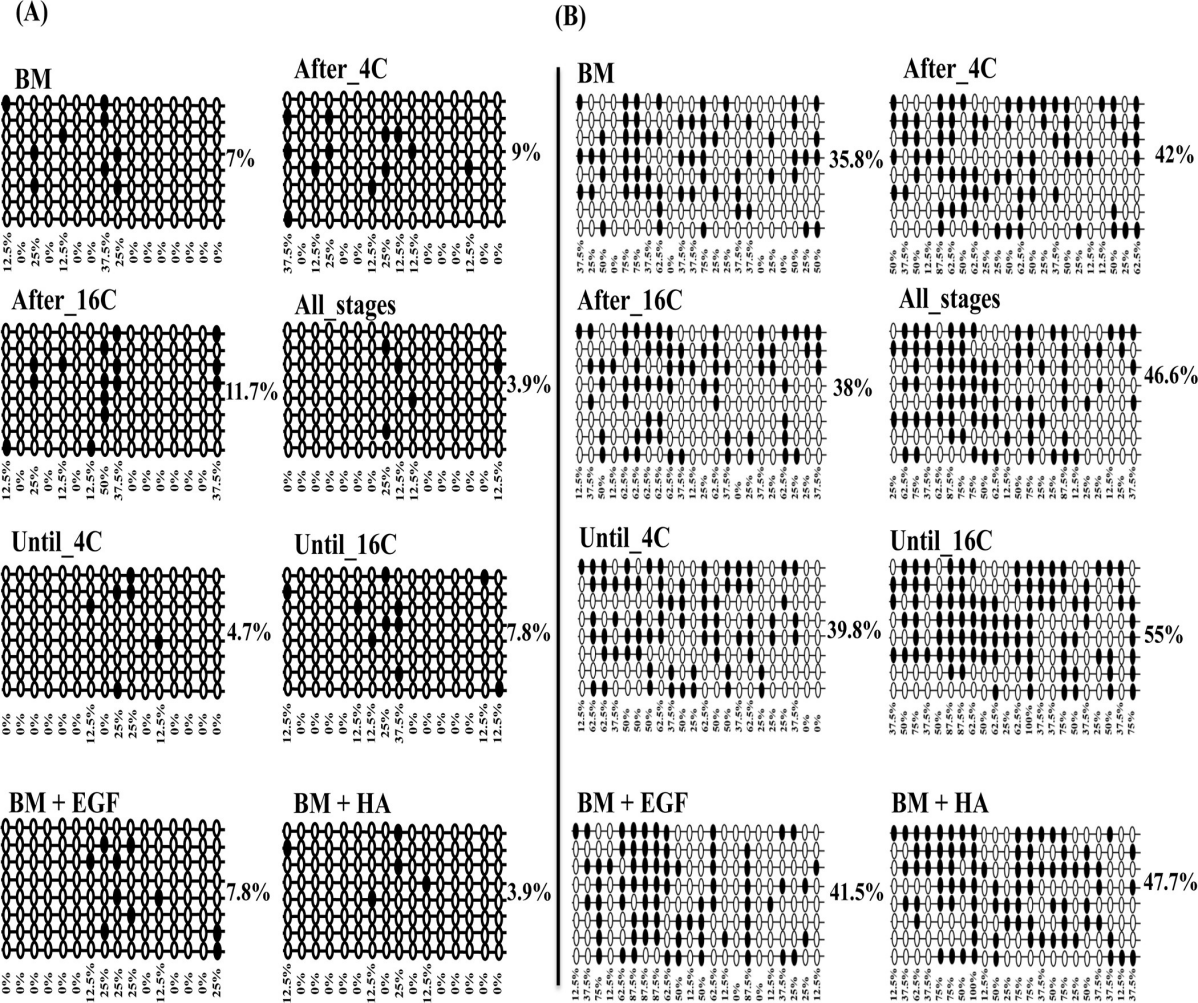
**The DNA methylation level of COL1A2 at the promoter (A) or distal promoter (B) region of blastocysts of different treatment groups**. The rows indicate the sequence of different colonies while, the circles indicate the CpG sites within the sequence The black and white circles indicate the methylated and unmethylated CpG sites, respectively.

Similarly, results showed that supplementation of HA alone or in combination with EGF through the preimplantation period decreased and increased the promoter and distal promoter DNA methylation level of COL1A2 gene in the resulting blastocysts. While, supplementation of EGF + HA after the 16-cell stage (After_16C) increased the DNA methylation at the promoter region of COL1A2 gene compared to Until_4C or Until_16C groups. However, higher DNA methylation at distal promoter region of COL1A2 was detected in blastocysts derived from Until_16C group (Fig 11).

**Fig 12:**
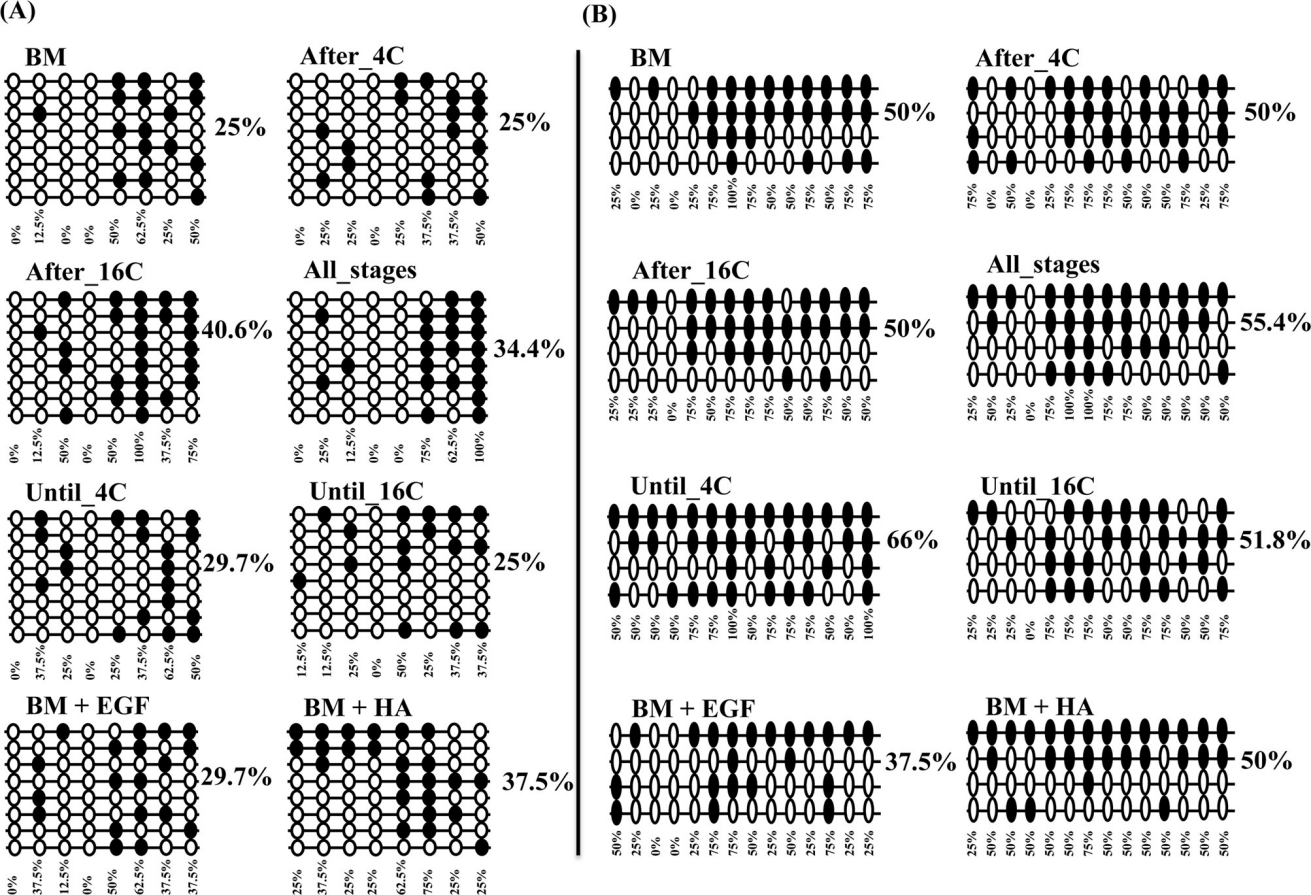
**The DNA methylation pattern of RAC1 gene at promoter (A) or gene body region (B) of blastocysts of different treatment groups**. The rows indicate the sequence of different colonies while, the circles indicate the CpG sites within the sequence The black and white circles indicate the methylated and unmethylated CpG sites, respectively.

In addition, the results showed that supplementation of embryo during the entire preimplantation period with EGF and/or HA increased DNA methylation level of RAC1 at the promoter region. However, EGF supplementation resulted in reduction of DNA methylation level at intronic region of the same gene. Moreover, the results from stage specific supplementation of EGF + HA showed that blastocysts obtained from After_16C group had the highest DNA methylation level at promoter region of RAC1 gene (Fig 12). At the intronic region of RAC1 gene, higher DNA methylation was detected in the blastocysts of the Until_4C group compared to blastocysts of all groups.

### Comparative analysis of gene expression and DNA methylation

Once, we have analyzed the expression and DNA methylation patterns in blastocysts of different groups, we performed comparative analysis between the expression and DNA methylation levels. For this, the DNA methylation data of COL4A1, COL1A2 and RAC1 were superimposed to the expression level. Accordingly, the results indicated that the DNA methylation pattern of COL4A1 at the promoter and distal promoter regions was negatively correlated with the gene expression patterns in blastocysts of EGF supplemented group. Moreover, at distal promoter of COL4A1, the DNA methylation pattern was negatively correlated with the corresponding gene expression in all treatment groups except After_16C, All_stages groups (Table 4). However, the DNA methylation level in the distal promoter region of COL1A2 was positively correlated with its gene expression in all blastocysts groups except in After_16 group. On the other hand, the DNA methylation of COL1A2 at the promoter region was negatively correlated with the corresponding gene expression in blastocysts of After_16C, All_stages, Until_4C and HA groups. Moreover, at the intronic region of the RAC1 gene, the DNA methylation pattern was positively correlated with gene expression in all blastocyst groups except After_16C, EGF and HA (Table 4).

## Discussion

Robust embryo-maternal communication permits normal development, high quality embryos, proper implantation and maintenance of pregnancy [24]. Thus, understanding the mechanism through which the preimplantation embryos communicate with *in vivo* or *in vitro* extracellular micro-environment is one of the most vital aspects to be considered in order to unravel the effects of suboptimal environmental conditions during embryogenesis. In this regard, our previous results demonstrated that, *in vivo* and *in vitro* alternative culture conditions altered the expression and DNA methylation pattern of genes involved in focal adhesion pathway in the resulted blastocysts [2, 3].

Focal adhesion is one of the essential molecular signaling pathways that regulate interaction and communication between the cell and the extracellular micro environment [25]. Focal adhesion is required for normal cell growth [26] and cellular functions [6, 25]. Indeed, previous studies indicated that normal cell growth is associated with stimulation of cell adhesion in the presence of growth factors [8]. In this regard, supplementation of epidermal growth factor modulated cell growth [27] and improved embryo development [28–34]. The potential effects of EGF may regulate by redistributing focal adhesion constituents to adhesion sites and by amplifying the levels of focal adhesion protein members [35]. Additionally, hyaluronic acid is an extracellular matrix component which is highly abundant in follicular, oviduct and uterine fluids of different species [36–39], is involved in multicellular functions [40–44] by increasing cell-to-cell communication and cell-to-extracellular matrix adhesion [45].Interestingly, supplementation of HA to *in vitro* culture media improved bovine and porcine oocyte maturation and embryo developmental competence [46–48]. Therefore, in the current study, bovine preimplantation embryos were in vitro cultured in the presence or absence of EGF and/or HA with to elucidate the potential effect of EGF and HA on bovine embryonic development and epigenetic landscape of genes involved in focal adhesion pathway. Results revealed that EGF and/or HA did not significantly affect the cleavage and the blastocyst rates. These results were in consistent with previous reports [19, 49, 50] which indicated that, EGF alone or in combination with HA did not influence the bovine blastocyst rate while other studies indicated that HA increased bovine blastocyst rate [46, 47, 51]. However, there was a positive effect of HA on reducing the percentage of apoptotic cells, suggesting the potential role of hyaluronic acid in regulation of cell proliferation and apoptosis compared to EGF group [44].

One of the critical cellular phenotypes that might mark the development and quality of *in vitro* produced embryos is the level of intracellular reactive oxygen species (ROS) [52]. In line to this, the current study revealed that EGF or HA significantly reduced the ROS levels in the resulted blastocysts. Indeed, EGF [53] and HA are believed to reduce the embryo intracellular ROS levels by regulating Nrf2 and Akt genes [54]. Nevertheless, the interaction between EGF and HA receptors is believed to increase the ROS levels [55]. Furthermore, cryo-preservability of the embryo is one of the main quality indicators of embryos developed under different culture conditions [56–58]. In the current study, HA was found to improve the blastocyst cryotolerance which was revealed by significantly higher blastocyst hatching rate at different time points after thawing. Previous studies also showed that, HA improved embryo cryotolerance and increased pregnancy rate after embryo transfer [13, 59, 60].

Following the phenotypic assessments, the effect of EGF and HA on the expression level of EGF receptor (EGFR) and HA receptors (CD44 and HMMR) was determined. EGF resulted in a significant decrease in the expression level of EGFR compared to other groups. These findings were in agreement with the previous studies which indicated that, excessive binding of EGF with its receptors led to reduction in mRNA level of EGFR [61]. On the other hand, a combined supplementation of EGF and HA induced the expression level of EGFR, this may suggest the potential role of HA in EGFR activation through specific receptors of HA namely, CD44 and HMMR [51, 62–65]. Moreover, CD44 is regulated by EGFR and the inhibition of EGFR resulted in a reduction of CD44 mRNA expression level [66]. In the present study HA induced the expression level of CD44 and HMMR receptors. HA is believed to promote the rapid appearance of HMMR expression [67] and the number of HMMR receptors increased significantly at morula stage of bovine embryos and then reduced when embryos are cultured in serum-containing medium [68].

The mRNA expression analysis revealed that, a combined supplementation of EGF and HA increased the expression level of candidate focal adhesion genes including the focal adhesion kinase (FAK) gene. FAK is a non-receptor tyrosine kinase focal adaptor protein which is localized in integrin-focal sites [69] to link the growth factors and integrins [35]. The expression of FAK is induced either by direct effect of growth factors such as EGF [35] or by the action of extracellular signal-regulated kinase1/2 (ERK1/2) which is activated by EGF and HA stimulation [35, 43]. The active FAK induces the phosphatidylinositol 3-kinase (PI3K) expression [69], ultimately leading to the induction of the RAC1 and PAK family genes [70, 71]. In line with this, our results revealed a higher expression level of RAC1 and PAK4 genes with the presence of HA. In fact, FAK and RAC1 are believed to induce focal adhesion complex proteins such as vinculin (VCL), a focal adhesion marker protein that controls focal adhesion [72, 73]. Our results also revealed a higher mRNA and protein expression levels of vinculin in blastocysts of EGF + HA compared to other groups.

Apart from this, considering the fact that major bovine EGA occurs around the 8- to 16-cell stages [74, 75] and the minor genome around the 4-cell embryonic stage of development, blastocysts were generated according to experimental design (Fig 1B). In this study there was absence of significant differences in embryonic development rates between all groups. On the other hand, the supplemented with EGF + HA from 4-cell stage until blastocyst stage (After_4C) or from zygote stage until 16-cell stage (Until_16C) increased the expression level of focal adhesion genes, indicating the importance of EGF + HA during the time of embryonic genome activation.

Additionally, the expression pattern of DNA methyltransferase genes (DNMT1, DNMT3A and DNMT3B) was investigated to get insight about the effects of EGF and/or HA on the expression pattern of genes involved in DNA methylation mechanism during embryonic development. The expression level of DNMT3A, which is one of the key genes involved in de novo DNA methylation, was significantly increased by the supplementation of EGF. However, it was significantly reduced with the supplementation of EGF + HA compared to control or HA groups. Similarly, previous studies also indicated higher expression of DNMT3A in bovine embryos cultured under different *in vitro* culture conditions [76]. On the other hand, the presence or absence of EGF + HA in culture media during the entire embryo genome activation period altered the expression level of DNA methyltransferase genes. These findings suggested that destabilization of embryos by the addition or removing of a single molecule particularly during entire genome activation period may lead to the disturbance of the DNA methylome of genes related to development. Therefore, we assumed that, the alteration of DNA methyltransferases particularly DNMT3A may influence the DNA methylation pattern of genes involved in focal adhesion pathway. For this, COL1A2, COL4A1 and RAC1 genes were selected for DNA methylation analysis based on their mRNA expression pattern. Bisulfite sequencing data revealed that the DNA methylation pattern of these candidate genes was changed by continued or stage specific supplementation of EGF and/or HA at the promoter, distal promoter or gene body regions. These results were found to be in agreement with our previous findings reported of *in vitro-in vivo* alternative embryo culture conditions [3]. Moreover, the DNA methylation level at the promoter regions of COL4A1, COL1A2 and RAC1 genes was induced in the EGF group compared to the HA one, but the gene expression tended to be lower in the EGF group compared to the HA group. Furthermore, the DNA methylation pattern of the candidate genes was inversely or positively correlated to the gene expression in the promoter, distal promoter or in the gene body regions. Previous studies reported a negative correlation between the DNA methylation level at promoter region and gene expression pattern [77] while, methylation at gene body correlates with gene activity [78]. Although our results did not show specific trend of the overall DNA methylation percentage of the selected genes however, the loss or gain of DNA methylation at specific CpG sites within the CpG island of candidate regions may result in different expression pattern of candidate gene. It was reported that, differentially DNA methylation patterns at a single CpG site could influence the transcription factors access leading to activation or inactivation of the gene [79, 80].

In conclusion, this was the first study that demonstrated the effect of culture media supplemented with EGF and/or HA before, during or after embryonic genome activation on development and quality of bovine preimplantation embryos, expression and DNA methylation pattern of genes involved in focal adhesion pathway. The presence of EGF and/or HA during bovine preimplantation embryo development altered the expression and DNA methylation patterns of genes involved in focal adhesion pathway associated with embryonic competence. Further studies are needed to investigate the effect of those components on embryo differentiated cells with the corresponding DNA methylome changes of specific CpG sites.

## Abbreviations

EGF: Epidermal growth factor
HA: Hyaluronic acid
DNA: Deoxyribonucleic acid
ECM: extracellular matrix
BM: Basic media
SOF: synthetic oviduct fluids
After_4C: Blastocyst group that was generated from zygotes culture in BM until 4-cell stage and then supplemented with 10 ng/ml EGF and 1 mg/ml HA (EGF + HA) until blastocyst stage
After_16C: Blastocyst group that was derived from zygotes cultured until 16-cell stage in BM followed by EGF + HA supplementation until the blastocyst stage
All_stages: Blastocyst group that was obtained from zygotes cultured until blastocyst stage in BM supplemented with EGF + HA
Until_4C: Blastocysts group that was obtained from zygotes cultured in BM supplemented with EGF + HA until 4-cell stage and further cultured until the blastocyst stage in BM
Until_16C: Blastocyst group that was generated from zygotes cultured in BM supplemented with EGF + HA until the 16-cell stage and then cultured in BM until blastocyst stage
HAmRNA: Messenger RNA
COCs: Cumulus oocyte complexes
PBS: Phosphate buffer saline
ROS: Reactive oxygen species
ICM: Inner cell mass
TE: Trophoectoderm
TUNEL: Terminal deoxynucleotidyl transferase (TdT)-mediated dUTP-biotin nick end labelling
qPCR: quantitative real time PCR
